# Physicians Report Barriers to Deliver Best Practice Care for Asplenic Patients: A Cross-Sectional Survey

**DOI:** 10.1371/journal.pone.0017302

**Published:** 2011-03-10

**Authors:** A. J. Jolanda Lammers, Joost B. L. Hoekstra, Peter Speelman, Kiki M. J. M. H. Lombarts

**Affiliations:** 1 Department of Internal Medicine, Academic Medical Center, University of Amsterdam, Amsterdam, The Netherlands; 2 Department of Quality and Process Innovation, University of Amsterdam, Amsterdam, The Netherlands; University of Giessen Lung Center, Germany

## Abstract

**Background:**

Current management of asplenic patients is not in compliance with best practice standards, such as defined by the British Committee for Standards in Haematology. To improve quality of care, factors inhibiting best practice care delivery need to be identified first. With this study, we aimed to identify and quantify physicians' barriers to adhere to best practice management of asplenic patients in the Netherlands.

**Methods and Principal Findings:**

A cross-sectional survey, preceded by multiple focus group discussions, was performed among Dutch physicians responsible for prevention of infections in asplenic patients, including specialists (of Internal medicine and Surgery) and general practitioners (GPs). Forty seven GPs and seventy three hospital specialists returned the questionnaire, yielding response rates of 47% and 36,5% respectively. Physicians reported several barriers to deliver best practice. For both GPs and specialists, the most frequently listed barriers were: poor patient knowledge (>80% of hospital specialists and GPs) and lack of clarity about which physician is responsible for the management of asplenic patients (50% of Internists, 46% of Surgeons, 55% of GPs). Both GPs and hospital specialists expressed to experience a lack of mutual trust: specialists were uncertain whether the GP would follow their advice given on patient discharge (33–59%), whereas half of GPs was not convinced that specialists' discharge letters contained the correct recommendations. Almost all physicians (>90%) indicated that availability of a national guideline would improve adherence to best practice, especially if accessible online.

**Conclusion:**

This study showed that, in accordance with reports on international performance, care delivery for asplenic patients in the Netherlands is suboptimal. We identified and quantified perceived barriers by physicians that prevent adherence to post-splenectomy guidelines for the first time. Better transmural collaboration and better informed patients are likely to improve the quality of care of the asplenic patient population. A national, online-available guideline is urgently required.

## Introduction

Patients without a spleen or with diminished splenic function are at risk of severe infections such as post-splenectomy sepsis (PSS), which carries a mortality of up to 70% [1], [2]. Patients can be protected from these infections if preventive measures such as immunizations and use of antibiotics are taken [3], [4].

As PSS is preventable, several relevant organizations have developed guidelines with recommendations for the management of patients without a spleen [5]. The British Committee for Standards in Haematology (BCSH) has developed a guideline for the management of patients after splenectomy in 1996 and updated it in 2002 [6], [7]. The recommendations such as made by the BCSH (see table 1) are currently thought to reflect ‘best-practice’ and physicians should adhere at least to similar practice. In the Netherlands, hospital specialists as well as general practitioners (GPs) are involved in the care for asplenic patients. During hospital admission, before and after splenectomy, specialists of Internal medicine and Surgery are responsible for immunizing patients and provide prophylactic antibiotics. After discharge from the hospital, patients are transferred to their GPs for further post-splenectomy management to prevent infections.

**Table 1:** Key recommendations for the management of asplenic patients by the British Committee for Standards in Haematology.

	Key recommendations
Immunization	Splenectomised patients should receive pneumococcal immunization (23-valent polysaccharide vaccine, PPV-23) and lifelong revaccination. They should also receive *Haemophilus influenzae* type B (Hib) and meningococcal C vaccine. Yearly influenza immunization is recommended.
Antibiotics	Continuous prophylactic antibiotics are recommended for the first two years after splenectomy. In case of suspected or proven infection during or after these 2 years, patients should immediately use antibiotics and be admitted to a hospital.
Education	All patients should be educated about the risks of infection associated with traveling (such as infection with *Plasmodium falciparum*) and unusual infections (i.e. dog bites). Patient records should be labeled to indicate the risk of infection as well.

Research on the quality of care for patients without a spleen has frequently reported low rates of physician guideline adherence [8]–[13], for instance in the United Kingdom, Scotland, Denmark, Spain and Canada. In the Netherlands, it was recently shown that management of patients after splenectomy is unsatisfactory as well [14], [15], and patients are therefore at risk of serious infection. A better understanding of the reasons for non-compliance with guidelines is needed to improve the care for asplenic individuals. Although low guideline adherence has been reported, to our knowledge potential reasons for this nonadherence have never been investigated. It is increasingly recognized that to improve guideline adherence, implementation should be preceded by the assessment of barriers [16], [17]. A wide range of potential barriers to guideline adherence has been identified operating at different levels, such as the level of the physician, of the patient, the organizational context and the social and cultural context [16], [18]–[20].

The aim of this study was to investigate barriers to comply with the best practice recommendations for asplenic individuals as made by the BCSH. We assessed reasons for nonadherence to recommendations for asplenic patients among general practitioners as well as hospital specialists and we identified the most important barriers that need to be addressed to improve adherence to best practice recommendations.

## Methods

We conducted a cross-sectional survey using written questionnaires, in a sample of Dutch general practitioners (GPs), and specialists of Internal medicine and Surgery. The survey was preceded by focus groups discussions to identify potential physicians' experienced barriers to adherence with best-practice (see Table 1) in the management of patients after splenectomy, as defined in the BCSH guideline [6], [7]. Since no human subjects were used and questionnaires were filled out by physicians anonymously, this study did not require an ethics approval, hence we did not contact the ethics committee of our institute.

### Questionnaire development

Conform state-of-the-art questionnaire development [21], we used focus-group discussions to gain insight into potential existing barriers to guideline adherence for GPs and medical specialists. Semi-structured focus group discussions were planned separately for GPs and hospital specialists (of Internal Medicine and Surgery). GPs were contacted by telephone, hospital specialists by email. Participants of different age, sex, years in practice and practice setting were selected.

We used the framework of Cabana and colleagues [18] to classify potential barriers to adherence to best practice recommendations into three main categories: barriers related to physicians' knowledge (i.e. lack of awareness and familiarity with the guideline), physicians' attitudes (i.e. lack of agreement, outcome expectancy or motivation) and external barriers (i.e. patient-, organization-, and guideline-related factors). These potential barriers were used to develop a topic guide. This topic guide, with open-ended questions, was then used to structure and moderate the discussion for each guideline recommendation. Each focus group discussion lasted two hours. Sessions were tape-recorded and fully transcribed. Two researchers (KL, JL) conducted an independent analysis of the transcript contents. Differences in interpretation of the transcripts were minimal and consensus was promptly achieved. Subsequently, the experienced barriers to guideline adherence that were identified during the two focus group discussions, were used in the development of two distinct questionnaires; one for GPs and one for hospital specialists.

The first part of the questionnaire was developed to determine current practice among the participating cohort of physicians. Each item had a 4-point Likert-type response: ‘always’, ‘frequently (in more than 50% of cases)’, ‘sometimes (in less than 50% of cases)’ or ‘never’. The second part of the questionnaire was designed to investigate experienced barriers to best-practice management, as outlined in the recommendations by the BCSH [6], [7]. For these items the 5-point Likert-type response was: 1 (strongly disagree), 2 (disagree), 3 (agree nor disagree), 4 (agree), and 5 (strongly agree). Suggestions for improving guideline adherence and respondents' demographics were requested in the third part of the questionnaire using closed questions. The questionnaires were pilot-tested before mailing them to the study sample. To obtain adequate response rates, we applied methodologies suggested in the literatures [22], [23], including factors such as length of questionnaire, traditional mail survey (as opposed to internet-based survey), providing return envelopes and follow-up mailing with replacement questionnaires.

### Study sample

The online database of the Academic Medical Center (AMC, Amsterdam) contains addresses of all Dutch GPs (ADB-ICT/Cluster Software Engineering, 2006 ADICT-AB, Amsterdam) and was used to randomly select a sample of GPs and obtain their contact details. Contact details of the sample of hospital specialists (Internists as well as Surgeons) were randomly selected from the “Geneeskundig adresboek Nederland”, where all Dutch specialists are registered. A total of one hundred GPs and two hundred hospital specialists (one hundred Internists and one hundred Surgeons) were invited to participate in the study. Questionnaires were sent with an accompanying informative letter and return envelope to all selected physicians. Two weeks after the initial mailing, a reminder card was sent to non-responders with the request to complete the form. Physicians received a second copy of the questionnaire if the first was not returned within 4 weeks.

### Data analysis

The results from all returned questionnaires were entered in a database. Data were analyzed with the Statistical Program for the Social Sciences (SPSS 16.0 for Windows®, SPSS Inc., Chicago, Illinois, USA). For analysis of demographic data, descriptive statistics were obtained. For the analysis of current practice, we categorized the answers ‘always’ and ‘frequently (in more than 50% of cases)’ as “positive”, and the answers ‘never’ and ‘sometimes (in less than 50% of cases)’ were categorized as “negative”. For analysis of statements about barriers given on the five-point scale, the answers were dichotomized to enable division between “yes” (barrier experienced) and “no” (barrier not experienced), by ranking *strongly agree* (5) and *agree* (4) as “yes” and *strongly disagree* (1) and *disagree* (2) as “no”. All p-values were computed by Chi-square test for three groups of physicians or types of work-setting by GraphPad (GraphPad Prism, version 4.00 for Windows, GraphPad Software, San Diego California USA).

## Results

### Study population

In total, 47 GPs and 73 hospital specialists participated in the study, yielding response rates of 47% and 36,5% respectively. One hundred and twenty questionnaires were suitable for analysis. Six questionnaires were excluded from analysis because they were returned without completion due to: no asplenic patients in practice (3), not engaged in care for asplenic patients (1), list too long (1), retired (1). The demographic characteristics of participating physicians are presented in Table 2.

**Table 2:** Characteristics of specialists (of Internal medicine and Surgery) and general practitioners (GPs) participating in the questionnaire survey.

	Internists	Surgeons	GPs
Number of participating physicians (N)	42	31	47
Mean age (years)	47	51	50
Gender (% male)	71	93	67
Mean years since registration as Specialist (MSRC registration)^a^	14,9	18,3	
Work setting (%):			
University hospital	14,3	26,7	
Non-university Teaching hospital	64,3	33,3	
General Non-teaching hospital	21,4	40,0	
Solo practice			30,4
Group practice^b^			63,0
Health center			6,5
Mean number of patients serviced by GP practice			2891

a MSRC = Medical Specialists Registration Committee.

b Group practice includes ‘duo-practices’ and ‘HOED-practices’ (Huisartsen Onder Één Dak; a number of GPs working independently in the same building).

### Current practice

Results of current practice are shown in Table 3. GPs and Specialists vaccinated their patients against pneumococci in 82,6% and 94,4% of cases respectively. Immunizations against *H. influenzae* B (Hib) and meningococci were given less frequently. The recommendation to take antibiotics immediately in case of fever was given in 90,5% of cases by Internists, in 60% of cases by Surgeons and 66% of cases by GPs. Continuous antibiotics for the first two years after splenectomy were prescribed in a minority (less than 15%) of patients by all physicians. These results indicate that current practice in the prevention of infections in asplenic individuals is not optimal.

**Table 3:** Current practice of asplenic patients' management as reported by specialists of Internal medicine and Surgery, as well as general practitioners.

Percentage of physicians reporting to provide asplenic patients with:	Internists (%)	Surgeons (%)	GPs (%)	P value^a^
Pneumococcal immunization	95,2	93,3	82,6	0,1123
*H. influenzae* B immunization	88,1	50,0	45,7	<0.0001
Meningococcal C immunization	81,0	56,7	30,4	<0.0001
Lifelong boosters of Pneumovax^b^	83,3	36,7	66,0	0,0002
Annual flu immunization	73,8	26,7	91,3	<0.0001
Continuous antibiotics for 2 years after splenectomy	9,5	13,3	6,4	0,5885
On-demand antibiotics	88,1	66,7	78,7	0,0887
Advice to take antibiotics immediately in case of fever	90,5	60,0	66,0	0,0123
Advice to gather information upon travelling	78,1	40,0	70,2	0,0026
Immediate antibiotic therapy after cat or dog bites	61,9	40,0	66,0	0,0648

Percentages indicate the number of physicians that answered with either ‘always’ or ‘frequently’ (in more than 50% of cases) when asked if they provided their asplenic patients with the recommended preventive measures.

a P value calculated by Chi-square test, for 3 groups of physicians.

b Pneumovax ®: 23-valent conjugate pneumococcal vaccination.

Comparison of different type of hospitals (university-, non-university teaching-, and general non-teaching hospitals) yielded no significant correlation between performance of hospital specialists and hospital teaching status (data not shown). Differences were minimal between types of GP-practices (solo practice, group practice and health centers) as well, although physicians working in solo-practices vaccinated their patients significantly less frequent against pneumococci (61,5%) and Hib (23,1%) as compared to GPs working in group practices (89,7% and 51,7% respectively) and health centers (100% for both vaccines) (data not shown).

### Knowledge related barriers

Physicians' knowledge of the recommendations for asplenic patients is shown in Table 4. Although not always familiar with the recommendations, less than 25% of hospital specialists and GPs indicated that they lacked sufficient awareness of the need for preventive measures in asplenic patients.

**Table 4:** Knowledge related barriers to best practice for asplenic patients.

Barriers	Internists(% agree)	Surgeons(% agree)	GPs(% agree)
**Familiarity**			
I am not familiar with the existence of recommended immunizations	4,9	33,3	37
I am not familiar with the existence of recommended ‘prophylactic’ and ‘on-demand’ antibiotics	55	70	71,7
I am not familiar with the existence of recommended precautions	29,3	58,6	55,3
**Awareness**			
I am not aware of the need for immunizations in asplenic patients	0	13,3	17,4
I am not aware of the need for antibiotics in asplenic patients	7,5	23,2	26,1

Percentages indicate the number of physicians that experienced the barrier, by answering either ‘strongly agree’ or ‘agree’.

### Attitude related barriers

Physicians' attitudes towards the recommendations for asplenic patients are shown in Table 5. Physicians generally agreed with guideline contents (65% of specialists, 73% of GPs) and evidence (58% of specialists, 68% of GPs).

**Table 5:** Barriers experienced by specialists of Internal medicine and Surgery, as well general practitioners.

	Internists (%)^a^			Surgeons (%)^a^			General practitioners (%)^a^		
Barriers	Vaccination	Antibiotics	Prevention^b^	Vaccination	Antibiotics	Prevention^b^	Vaccination	Antibiotics	Prevention^b^
**Attitude-related barrier**									
I do not agree with the guideline contents	7,1	10,5	4,9	0	20	3,3	2,2	4,3	6,4
Recommendations are not evidence-based	9,5	15,4	12,2	0	13,3	0	2,2	4,3	8,5
Recommendation is time consuming	14,3	2,6	-	0	3,3	-	4,3	4,3	4,3
Patients' comorbidity	42,9	-	-	17,2	-	-	-	-	-
Long-term use of antibiotics is a patient burden	-	-	-	-	-	-	-	43,5	-
**External factors**									
Physicians' responsibilities are not clarified	53,5	44,7	51,2	43,3	46,7	48,4	63	60,9	40,4
The specialty registrar^c^ is not aware of the need	61,9	56,4	51,2	50	53,3	48,3	55,3	54,3	51,1
The patient is not informed about the need	83,3	81,6	-	90	90	-	80,9	89,1	-
Patient is resistant to receive the measure	16,7	20,5	-	13,3	20	-	23,4	28,3	-
The GP does not comply with my suggestion	33,3	59	58,5	40	41,4	41,4	-	-	-
The specialists' instructions are incorrect	-	-	-	-	-	-	32,6	45,7	27,7
The specialists' instructions in the discharge letter are incomplete	-	-	-	-	-	-	46,8	52, 2	51,1
Different hospitals recommend different policies	-	-	-	-	-		31,9	45,7	36,2
Lack of reimbursement for NeisVac-C vaccin	-	-	-	-	-	-	25,5	-	-

a percentages indicate the number of physicians that either “strongly agree” or “agree” with the proposed barrier.

b prevention: give advice to patient when travelling and prompt treatment of unusual infections.

c specialty registrar = in training for Medical or Surgical consultant, general practitioner in training.

### External barriers

Several barriers related to patient- and organizational levels were experienced (Table 5). According to respondents, adherence to the recommendations would improve if patients themselves would be better informed (over 80% of hospital specialists and GPs). Physicians reported barriers on organizational level to be important as well: clarity about which physician is responsible for the management of asplenic patients was lacking (50% of Internists, 46% of Surgeons, 55% of GPs), and physicians were not able to rely on their residents in the management of asplenic patients (over 50% of hospital specialists and GPs). Moreover, hospital specialists were uncertain if the GP would follow their advice given on patient discharge (33–59%), whereas GPs were not convinced that the specialists provided them with a discharge letter containing the correct recommendations (47–52%).

Differences in these perceived barriers between specialists working in different hospital types were minimal. Medical specialists working in university hospitals were most confident in their residents (41,2%, as opposed to 14,1% of Specialists in non-university teaching hospitals, and 11,1% in non-teaching hospitals). Surgeons working in general non-teaching hospitals especially reported lack of clarity on transmural responsibilities as a barrier (48,5%, compared to 17,2% of surgeons in non-university teaching hospitals, and 8,3% in non-teaching hospitals) (data not shown).

Lack of clarity on responsibilities as well as incorrect discharge letters were reported most frequently by GPs working in health centers (both 44.4%), as compared to GPs working in GPs working in group practices (19,5% and 14,9% respectively) or solo GPs (10% and 5%). Solo GPs reported the lowest confidence in residents (2,4%, as compared to 10,3% of GPs working in group practices and 33,3% of GPs working in health centers) (data not shown).

### Improving adherence to recommendations

**Figure 1:**
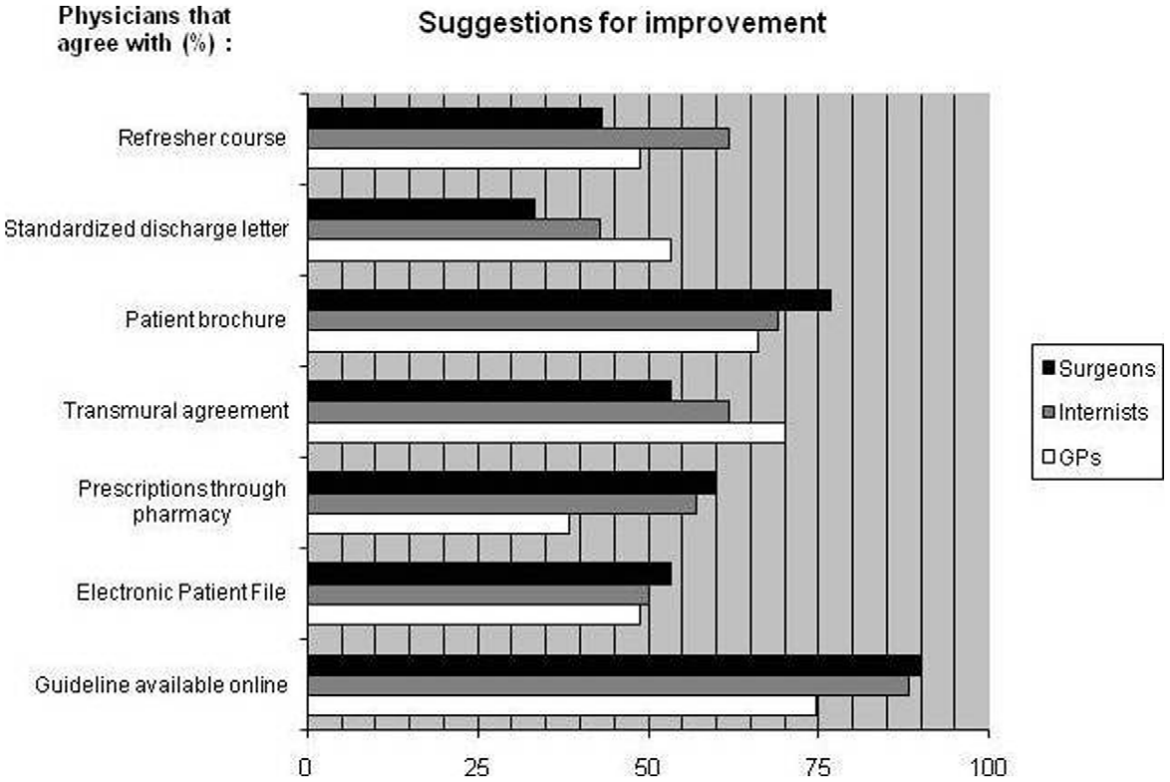
Potential improvements in the adherence to recommendations for asplenic patients. Percentage of general practitioners (GPs), Specialists of Internal medicine and Surgery that indicated the suggestion as potentially useful.

All physicians indicated that the availability of a comprehensible national guideline containing all recommendations would improve adherence to best practice (93% of Internists, 94% of Surgeons and 97% of GPs, data not shown). Respondents scored the predefined potential improvements for (better) compliance with best practice recommendations. Results are shown in Figure 1. Of all given suggestions, the respondents rated the online availability of a guideline as most useful. Other suggestions for improvement, identified during the focus group discussions, that were regarded as useful by most respondents were ‘a patient brochure’ (69,8%) and ‘transmural agreements about responsibilities’ (63,3%).

## Discussion

### Main findings

Current management of asplenic patients in the Netherlands is not in compliance with best practice standards. This study identified, for the first time, the barriers which inhibit physicians' adherence to recommendations for asplenic patients. The study revealed that in the Netherlands, problems on an organizational level and poorly informed patients most likely explain non-adherence of physicians to the best practice recommendations.

### Explaining the results

This study used physicians' self-reporting to measure current practice of post-splenectomy management of patients. The reported findings are in correspondence with our previous study [14], where comparable vaccination- and antibiotic prescription rates were found. Internationally, publications report comparable numbers as well [9], [12], [13], [24]. Maybe the most alarming finding is that GPs as well as Surgeons fail to recommend well over one third of all asplenic patients to take antibiotics immediately in case of infection, which is the most important measure to prevent lethal infections in asplenic patients.

For improvement purposes we identified physicians' barriers to best-practice after splenectomy which we classified using Cabana's framework [18]. In this study we found that poor adherence to the British guideline recommendations is probably not due to lack of knowledge or negative attitude towards the guidelines. Physicians' knowledge of the guideline recommendations for asplenic patients was generally adequate. Although not always familiar with the recommendations, less than a quarter of hospital specialists and GPs indicated that awareness was a barrier to adherence. This is in correspondence with reported figures from Canada, were physicians' knowledge of management of asplenic patients was also found to be relatively satisfactory [25]. In our study, general practitioners estimated to serve a mean of 3,3 asplenic patients per 2500 patients in their practice (data not shown). Therefore, although prevalence is low, it is encouraging to see that knowledge of the infectious risks of these patients is fair. Further, physician's attitudes towards the recommendations for asplenic patients were generally positive. Physicians reported to have confidence in the guideline contents. In addition, despite the fact that many BCSH recommendations were formulated based on expert opinion (low level of evidence), physicians indicated they found the evidence to be sufficient.

Appropriate knowledge and attitudes of physicians are necessary but not sufficient for adherence to guidelines [18], [26]. A physician may still encounter barriers that limit his/her ability to perform the recommended behavior due to so called external factors, that is patient related, guideline related or organizational factors.

Indeed, we found that Dutch physicians experienced external factors to be important barriers to adherence. Organizational factors, such as lack of clarity on the responsibilities in care delivery for asplenic patients were reported to be most important. In the Netherlands, care for asplenic patients is a joint responsibility of specialists (during hospital stay) and general practitioners (after the patient has been discharged from the hospital or outpatient clinic). Both groups of physicians indicate that clarity about the division of transmural tasks most likely would improve care for asplenic patients. There is also a lack of mutual trust: hospital specialists are uncertain whether GPs will follow their recommendations after discharge, whereas at the same time the GP is not confident that the referring consultant has provided a discharge letter containing complete and correct recommendations. Clearly, here is room for improvement, when both groups of physicians reach consensus on the responsibilities of implementation of the recommendations for asplenic patients. In addition, both GPs and hospital specialists indicated that better informed patients may contribute to improved quality of care. Although patient education alone is not sufficient to prevent post-splenectomy sepsis, it may be an important factor in preventing infections during asplenia. El-Alfy *et al.* studied 318 patients after splenectomy [27], and found that 45% was well informed about the risk and prevention of infection, 30% had fair knowledge and 25% had poor knowledge. Patients displaying greatest knowledge had significantly lower prevalence of PSS as compared to those with poor knowledge (1,4% versus 16.5%, p<0,001).

### Limitations

Physicians lacking knowledge or affinity regarding asplenia might have been more reluctant to participate in the study, thereby inducing a positive bias. Furthermore, response rates were modest, 47% of GPs and 37% of hospital specialists. This could induce a sampling bias as well. Lastly, although we did find that there was strong agreement amongst the different groups of physicians on perceived barriers; low responses could also negatively influence accuracy of the results.

This survey however had a solid research design as both qualitative and quantitative methods were used. The qualitative approach enabled optimal exploration of reasons for nonadherence, after which the identified barriers were quantified in the cross-sectional study. Overall, our results have clear implications for initiatives to improve physician adherence in order to optimize care for asplenic patients.

### Implications for practice and research

This survey provides an overview of the range of barriers that prevent physician adherence to post-splenectomy guidelines. Issues that should be addressed according to Dutch hospital specialists and GPs are: improving patient education and increase clarity on responsibilities and implementation of care for asplenic individuals. Education of health care professionals and patients regarding the risk of infection after splenectomy remains a must, perhaps more important than chemoprophylaxis, immunoprophylaxis or any specific surgical intervention. Therefore, the development of a Dutch guideline is urgently required.

### Conclusions

This study showed suboptimal care delivery for asplenic patients and both identified and quantified physicians' experienced barriers to comply with best practice recommendations for the management of post-splenectomy. Better informed patients and better transmural collaboration between GPs and hospital based Internists and Surgeons are likely to improve the quality of care of the asplenic patient population.
